# What do patients want from their psychiatrist? A cross- sectional questionnaire based exploratory study from Karachi

**DOI:** 10.1186/1471-244X-8-14

**Published:** 2008-02-29

**Authors:** Roomasa Channa, MN Siddiqi

**Affiliations:** 1 Medical College Aga Khan University Hospital, Karachi, Pakistan; 2 Department of Psychiatry Aga Khan University Hospital, Karachi, Pakistan

## Abstract

**Background:**

The aspects of consultation that are important for psychiatric patients have always remained a less acknowledged area. The aim of this study was to identify these aspects.

**Methods:**

A Cross-sectional, questionnaire based study was carried out in a psychiatry outpatient clinic of two tertiary care hospitals in a developing country. The patients were asked to fill out the questionnaire containing a total of 11 close-ended questions plus 1 open-ended question. They graded them as not important, important, very important or do not know. Non-psychotic patients aged 18 and above, visiting the clinic were recruited into the study before they went in for their first consultation.

**Results:**

The response rate of patients was 84%. More than 90% wanted the doctor to tell them the cause of their illness, talk to them about their condition, provide symptomatic relief, let them know that how long their illness would last and make the final decision about their treatment plan. Less than 20% wanted to be part of a support network. A significant 82% wanted talking therapy as part of their treatment plan.

**Conclusion:**

The three issues, most important for patients were: the doctor should listen to them, make the final decision about treatment and provide symptomatic relief. Only 20% wanted to be a part of patients' support group.

## Background

Studies have shown that patients' level of satisfaction improves when their perspective is taken into account during the consultation [1,2]. However little is known about the issues which may be important to psychiatric patients from developing countries like Pakistan. It is also known that patients' primary concerns usually differ from the physicians' agenda and often go undetected [3]. Therefore, it becomes even more important to understand the issues important in our setting in order to avoid patients' dissatisfaction, non-compliance with recommendations and premature termination of therapy [4].

Psychiatrists should encourage patients to verbalize the issues important to them, which should then be incorporated as much as possible in the treatment plan [4]. Studies have identified a positive relationship between this approach and patient satisfaction and re-attendance [1,2]. There are 150–200 qualified psychiatrists for a population of 150 million in Pakistan [5]. Majority of them are urban-based [6]. Many practice in Karachi, the largest city of Pakistan, having a population of 15 million [7]. Thus, patients from far off areas of the country have to come to bigger cities like Karachi to avail the psychiatric services. Prevalence of common mental illness is high in Pakistan [8,9] as is poverty [10] while literacy rate is low [11]. Stigma attached to these patients is strong [12,13]. Studies show that considering patients' views becomes particularly important in the psychiatric setting where the service users are often socially and economically marginalized [14]. Issues important to them may be sidelined unless psychiatrists make a conscious effort to explore them.

Although similar studies have been carried out in the West [14-16], data from Asia is conspicuously lacking. We do not know if similar issues are as important to our patients. Due to cultural differences, relying on western studies might not be appropriate. If psychiatrists know the issues important to their patients, they may be able to endorse this knowledge, incorporate it into their management plan and provide care that is more meaningful. Early determination of issues important to patients could therefore be the first step towards provision of an effective and meaningful care.

## Methods

This study was a cross-sectional questionnaire based exploratory survey that was carried out in two tertiary care teaching hospitals of Karachi, Pakistan. These hospitals are postgraduate training centres, each having a capacity of around 500 in-patient beds. Locally and internationally trained psychiatrists conduct daily clinics here. In Pakistan, there is no concept of different tiers and no system of proper referral to any hospitals. There is also no separate mental health care system. There are in fact no gatekeepers. Patients come from different sources, for example, general practitioners, faith healers, and some are referred by other psychiatrists for a second opinion. However, the main bulk of these patients approach these facilities by themselves, are referred by other physicians or visit on the recommendation of other patients' or their own relatives [17]. As there is no specific catchment area for a particular hospital, patients come from all over the city and from far off areas of the country. This study involved recruiting consecutive patients as they walked into the clinic but before their consultation with the psychiatrist.

The study was conducted over a period of three weeks i.e. from 15^th ^November to 6^th ^December 2006.

### Inclusion Criteria

Non-psychotic patients aged 18 and above, coming for the first time to psychiatric out-patient clinics for consultation.

### Exclusion criteria

Psychotic patients, patients under 18 years of age, follow-up patients and patients who refused to give consent.

Despite extensive literature search, we were unable to find a previously validated questionnaire that would adequately assess our objective of determining issues important for psychiatric patients in our setting.

We therefore self constructed the questionnaire using psychiatric patients, their accompanying relatives, staff of psychiatric out patient clinics/wards and trainee psychiatrists as our sources. Patients were asked which issues were important to them when coming for their first consultation. Others were asked open-ended questions about what expectations and issues, patients generally had regarding their treatment plan. Based on the most commonly identified issues, a questionnaire was constructed, which had 11 closed-ended questions and 1 open-ended question. (See Additional File 1)

Patients were asked to answer the 11 questions by grading them from 1–4; with 1- not important, 2-important, 3- very important and 4- do not know. An open-ended question was included so that, patients could point out anything else which may have been important but was not addressed in the questionnaire. Out of 50 respondents, only 7 answered this part of the questionnaire. We expected that it would help to identify those issues, which we may have missed out.

The questionnaire was first written in English and then translated into the local language, Urdu, by an expert of both languages. Back translation was done to further verify the accuracy of the translation. The final questionnaire had both languages written together. There were three illiterate patients, to whom the questions were read out but no explanation was given.

The patients were first given an informed consent form and then the questionnaire. This form was also written in both languages- English and Urdu. It stated the nature and objective of the study with a clear statement that participation or refusal to participate would not affect their rights of care in any form. If they refused, they were excluded from the study. The questionnaire was handed to those who agreed to sign the consent form. The participants were required to fill questionnaires before starting their consultation with the psychiatrist. The total response rate was 84%

We performed a pilot study on five patients, which helped to decrease ambiguity in the questionnaire.

The Ethical Approval for our study was obtained from Departmental Ethical Committee for Research, Department of Psychiatry Faculty Office Building Aga Khan University Karachi Pakistan.

## Results

Fifty out of 58 patients approached, responded. There were 26 males and 24 females. Of these 25 were employed and 25 were unemployed. The average age of the patients was 33 years (± 13.1 years), ranging from a minimum of 18 years to a maximum of 73. The questionnaire asked about the income status, but this variable had 13 missing values, as 26% of the patients did not respond. There were 94% literate patients, with 82% having secondary to graduate level education. Application of chi-squared test revealed that p-values for all our variables were more than 0.05. We did not find an association between the demographic factors and the different issues identified. The percentages give the proportion of patients for whom that particular issue was either important or very important (See Table 1)

**Table 1:** showing the items in the questionnaire and the proportion of patients, with confidence intervals. The items below all start with the question: "How important is it for you that the doctor should...?"

Items in the Questionnaire	Proportion of patients	Confidence intervals
Let you talk about your condition	96%	90.6% – 100%
Discuss treatment options and DOCTOR makes the final decision	96%	90.6% – 100%
Provide symptomatic relief	96%	90.6% – 100%
Tell the patient how long the illness will last and the number of follow-ups	94%	87.4% – 100%
Explain the cause of your condition	92%	84.5% – 99.5%
Inform you about side effects of treatment	86%	76.4% – 95.6%
Discuss inclusion of talking therapy in the treatment plan	82%	71.4% – 92.6%
Discuss treatment options and YOU make the final decision	65.3%	51.8% – 78.2%
Discuss cost of medicines	58%	44.3% – 71.7%
Order laboratory test	44%	30.2% – 57.8%
Make you part of a support network that includes other patients like you	20%	8.9% – 31.1%

Seven out of a sample of fifty patients (14%) responded to the open-ended question. Respondents did not uniformly point out any one particular issue. For example, one mentioned that maintaining communication with the doctor over long distance was important for him, as he had travelled across the country for the consultation. Another mentioned confidentiality of the diagnosis, et cetera.

The three issues most important for the patients were that the doctor should listen to them, make the final decision about treatment options for them and provide symptomatic relief. There were 96% patients who responded by saying that the above issues were either important or very important for them and 65.3% of the patients said that they would like to make the final decision after the doctor had explained to them the pros and cons of different treatment options. However, for 96% of the patients, it was important that the doctor should make the final decision. This suggests an overlap between the two groups. Ninety-four percent of the patients expected to be informed about how long their illness would last and 92% were interested in knowing the cause of their illness. 86% of our patients were interested in knowing the side effects of their treatment and 82% patients felt that non-pharmacological treatment was important for them. Only 8% did not know about such treatment option. The cost of medicine was either important or very important for 58% of participants. Less than half i.e. 44% of the patients considered it important to get the laboratory tests done. Forty-two percent replied with a "do not know" for this question on laboratory investigations. Only 20% of the patients wanted the doctor to introduce them to a support network whereas 10% answered with 'do not know' and a significant 70% answered that it was not important for them to be part of a support network.

## Discussion

The issue of 'listening' by doctors' during consultation can be due to communication or shortage of time, which is universal. Perhaps symptomatic relief comes under the same category. This issue however needs further elaboration so that we can determine the kinds of symptoms that are most distressing for them. In the western world a great deal of emphasis is given to the patients' choices and empowerment of patients in the decision making process [18]. In spite of the fact that our patients still rely on the doctors to give the final decision, they want to play an active role in the decision making process. This finding becomes even more interesting when seen in the context where a doctor is still looked upon as someone who knows best and is expected to make health related decisions. Perhaps it reflects a changing trend towards more empowerment of patients but we do not have a local study with which to compare our findings. Therefore, this is purely a speculation based upon a general impression of changing values of the society.

An overwhelming proportion of patients wanted to know how long their illness would last. This question might be ambiguous. It is related not only to the length of illness but also to the number of follow-ups. The implications of the two may be different. Therefore, the response to this question may not be a true reflection of their thoughts.

It is easier to comprehend that the patients want to know the cause of their illnesses but a minority in our study did not consider it important. The sample size is too small to determine which particular characteristics are common in such patients who do not wish to know the cause of their disease. Similarly, a minority also felt it unimportant to know the side effects of the treatment. One wonders whether they felt more uncomfortable about knowing the side effects, were afraid of its effects on the compliance or were so distressed by the disease that they were willing to pay any price to get rid of the illness. Future work may however help us to understand this issue. A study showed that 76.2% of outpatients coming to a general physician wanted to know all possible adverse effects [19]. Their result matches our own finding.

A review suggested that patients most commonly request psychological interventions [4]. Our study replicates this finding. Patients do want psychological interventions but psychiatrists often fail to identify what their patients' want [18]. This may lead to patient dissatisfaction. A study, in which patients rated the expected helpfulness of 14 interventions, reported that psychological interventions were most helpful [2].It is important for the psychiatrist to strike a balance between psychotherapy and pharmacotherapy. It has been seen that patients treated with a combination of both are more satisfied [20].To meet this demand in a country where psychiatrists are already scarce, would be a challenge.

Cost of treatment is becoming an issue even in the developed world [21]. It is going to be more important in Pakistan where, about 70% of the population earns less than $2 per day [22].The issue of cost was highlighted by doctors and patients during discussions, prior to construction of the questionnaire. The importance of cost should also be seen in the context of unemployment, minimal state sponsored health facilities, lack of insurance for health care and relatively longer course of treatment for mental illnesses. Hence, the finding that 58% of participant marked the cost as "important" to them was not as surprising as was the fact that 34% marked it as "not important". One explanation could be that setting of study was in private hospitals. Therefore, relatively affluent sections of society visit these clinics and for them cost may not be an issue. On the other hand, a proportion of poor patients might have marked it unimportant due to the fear that the doctor may prescribe cheaper but less effective medications, if constrained by cost issues. Until we have a larger and more representative sample, we will not know the rationale behind these responses. However, even this study does show that cost is a real issue for majority of our patients. Further exploration of this finding would be interesting.

A significant proportion of respondents felt that laboratory investigations were not important. It is possible that they had already ruled out organic causes of their disease before coming to a psychiatrist. It is noteworthy that almost similar number of patients did not know whether it was important or not to have laboratory investigations. Perhaps this is an area where they were much less knowledgeable and felt the doctor should take the lead. This makes sense if seen in the background of the above finding, where patients wanted the doctor to make a final decision.

Social support is important for effective management of psychiatric patients [23,24]. Social support comes from different sources that include family, friends and formal groups of patients [24]. The culture of support groups has still not been developed in Pakistan; on the other hand, family structure is disintegrating. Against this background, patients' support groups might provide a useful resource. We were interested in finding how many of our patients wanted to be a part of such a support network, and we discovered that majority of our patients did not want to be a part of a network. We do not know if they were concerned about issues of confidentiality, did not grasp the concept, did not need extra support because the current support was sufficient or thought that fellow patients may be sympathetic but will not be able to provide the required support as seen in earlier studies [25]. Until we know the reasons behind this response, we would not be able to comment on what type of support will be more acceptable in our culture.

A few studies have examined links between patient variables and different issues important to them. Being older or being a female has been associated with favourable outcome about treatment's helpfulness [26]. In our study, we did not find any significant association between the different issues identified and patient variables like age, sex, income, employment status or level of education. On application of chi-squared, all p values were more than 0.05

There are many limitations in our study. Small sample size could explain our failure to detect any significant association between the importance of various issues and different demographical variables.

Although a pilot study was performed on five patients, to decrease ambiguity in the questionnaire and to provide a validity check, we do not know the psychometric properties, validity, factor structure or Cronbach's alpha of the questionnaire

We have already discussed that question number 4 was ambiguous because it does not clarify whether we were enquiring about the length of illness or number of follow-ups. Furthermore, this is the only question where instead of addressing the respondents directly, it asked their view in third person. This mistake would need to be rectified in future study.

Non-responders, constituted 14% of the total population. Comparison with responders could have given us useful information. However when we approached patients, we asked about their willingness to participate and if they refused we did not proceed with any questions. We could have gathered their demographic details from the registration booth but we did not do so. Therefore, this comparison could not be made, we cannot comment on the characteristics of the non-responders and a selection bias cannot be ruled out.

Issues that are important for patients', prior to contact with the service, may change once the treatment process has begun [18]. Therefore, we included only those patients coming to this particular clinic for the first time. However, we did not establish if some of them had already visited other clinics thereby having different expectations compared to the "true" first time attendees. Neither did we know the source of referral, which could have also influenced expectations. Similarly, the nature, duration and severity of illness could also affect patients' expectations. We did not know the diagnoses, duration of illness or the presenting symptoms, all of which are important in shaping or modifying expectations.

The study was conducted at two large teaching private hospitals. The fee charged by these hospitals is likely to affect the study in at least two ways: a) the lower socioeconomic strata may not able to afford the facilities and therefore the results cannot be generalised, b) patients who pay the fee may have a different perspective about a service or consultation compared to those who avail the services free of cost.

We may not be able to address all the issues important to our patients, yet knowing what is important for them is likely to help in chalking out a more effective, patient oriented management plan. This prior knowledge becomes particularly important in areas where services are scarce, few trained psychiatrists are available and time has to be divided among many patients. Based on the limitations of this study, the scientific value of the study may not be significant, yet this study does highlight an important but unexplored clinical area. Furthermore, this is an exploratory study and is the first step to understanding issues important for a section of the Pakistani population. Further research with robust scientific measures, larger sample size and inclusion of unexplored issues is likely to enhance our understanding of this important clinical area and its potential applicability in developing countries like Pakistan.

## Conclusion

It was important for most patients that the psychiatrist listened to them, explained the cause of their illness, made the final decision about treatment options and provided symptomatic relief to them. Only a small proportion wanted to be part of a support network. We did not find any significant relationship between the various issues and patient factors like age, sex, employment status, income and educational background. This exploratory study has raised many questions and highlighted many issues that need further exploration.

## Competing interests

The author(s) declare that they have no competing interests.

## Authors' contributions

**RC**- Study conception, data collection, entry and analysis, manuscript writing

**MNS**- Study conception, manuscript writing, revision, editing and overall supervision.

Both authors have read and approved the final manuscript.

## Pre-publication history

The pre-publication history for this paper can be accessed here:



## Supplementary Material

Additional file 1 questionnaire. The actual questionnaire as used in this study. Click here for file
