# Supplementary material for: What do patients want from their psychiatrist? A cross- sectional questionnaire based exploratory study from Karachi
Source: BMC Psychiatry. 2008 Feb 29;8:14. doi: 10.1186/1471-244X-8-14 (PMC2275251; doi:10.1186/1471-244X-8-14)
Supplement: Additional file 1 — questionnaire. The actual questionnaire as used in this study. [file 1471-244X-8-14-S1.doc]

Age:

Sex:

Educational Background:

Occupation:

Monthly Family Income:

----- Less than 20,000 -----20,000-40,000 -----40,000-60,000 -----more than 60,000

Please answer the following questions by grading them according to:

1(not important) 2(important) 3(very important) 4 (don’t know)

How important is it for you that the doctor should…?

| 1 | 2 | 3 | 4 |
| --- | --- | --- | --- |
| 1 | 2 | 3 | 4 |
| 1 | 2 | 3 | 4 |
| 1 | 2 | 3 | 4 |
| 1 | 2 | 3 | 4 |
| 1 | 2 | 3 | 4 |
| 1 | 2 | 3 | 4 |

1. Doctor should explain the cause of your condition

2. Should let you talk about your condition

3. Make you part of a support network that includes other patients like you

4. Discuss treatment options and YOU make the final decision

5. Discuss treatment options and DOCTOR makes the final decision

6. Provide symptomatic relief

7. Inform you about side effects of treatment

8. Discuss Alternative treatment (that is other than medicines, for example talking

therapy

| 1 | 2 | 3 | 4 |
| --- | --- | --- | --- |
| 1 | 2 | 3 | 4 |
| 1 | 2 | 3 | 4 |
| 1 | 2 | 3 | 4 |

9. Order laboratory tests

10. Tell you how long the illness will last and the number of follow-ups

11. How important is it for you that the doctor should discuss the cost of medicine

12. Please add anything else that you think is important but has not been asked in the questionnaire
